# Collagen cross-linking as monotherapy in experimentally induced corneal abscess in rabbits

**DOI:** 10.1186/s12886-023-03007-y

**Published:** 2023-06-13

**Authors:** Zeinab A. Saad, Hazem Elnashar, Sahar Negm, Hala A. Elsayed, Mohamed Gaber Abdallah, Tamer M. M. Abuamara, Wagih M. Abd-Elhay, Hanan M. Elghonemy

**Affiliations:** 1 Memorial Institute of Ophthalmic Research, Giza, 12511 Egypt; 2 Research Institute of Ophthalmology, Giza, Egypt; 3 Faculty of Medicine, Department of Medical Biochemistry, Al-Azhar University, Cairo, Egypt; 4 Faculty of Medicine, Histology Department, Al-Azhar University, Cairo, Egypt

**Keywords:** Collagen cross-linking, Microbial keratitis, *Fusarium solani*, *Pseudomonas aeruginosa*

## Abstract

**Background and purpose:**

Collagen cross-linking (CXL) has evolved as an essential therapeutic approach for corneal infections, allowing for rapidly eliminating the infecting microorganism while reducing inflammation. This study aims to evaluate the efficacy of CXL as a monotherapy for managing infectious keratitis caused by *Fusarium solani* and* Pseudomonas aeruginosa.*

**Materials and methods:**

Forty-eight white New Zealand rabbits weighing approximately 1.5–2 KG were included. The cornea of one eye of each rabbit was inoculated with either *Fusarium solani* or *Pseudomonas aeruginosa*. Group A served as a control and was subdivided into two subgroups, A1 and A2; each subgroup consisted of 8 eyes and was injected with either *Fusarium solani* or *Pseudomonas aeruginosa,* respectively. Group B (16 eyes) was inoculated with *Fusarium solani*, while group C (16 eyes) were inoculated with *Pseudomonas aeruginosa*. All animals in Group B and C received CXL treatment one week after inoculation of the organisms and after corneal abscess formation was confirmed. At the same time, animals in Group A were left untreated.

**Results:**

There was a statistically significant reduction in the number of colony-forming units (CFU) in Group B following CXL. No growth existed in any samples at the end of the 4th week. There was a statistically significant difference in the number of CFU between group B and the control group (*p* < 0.001). In group C, there was a statistically significant reduction in the CFU at the end of the first week after CXL. However, there was regrowth in all samples afterward. All 16 models in Group C showed uncountable and extensive growth during the subsequent follow-ups. There was no statistically significant difference between the number of CFU in Group C and the control group. Histopathology showed lesser corneal melting in CXL-treated *Pseudomonas aeruginosa*.

**Conclusions:**

Collagen cross-linking is promising monotherapy and alternative treatment in managing infective keratitis caused by *Fusarium solani* but is less effective in *Pseudomonas aeruginosa* as monotherapy.

## Background

The corneal abscess is a destructive and sight-threatening corneal disease that can severely affect vision. Treatment with topical antibiotics or even keratoplasty in advanced cases is the conventional way of management [1]. Furthermore, progressive cornea melting due to collagen breakdown may result in perforation in advanced cases; hence anti-inflammatory and anti-collagenase drugs are frequently needed [2].

Corneal cross-linking (CXL) is a photochemical reaction that employs ultraviolet A (UVA) light and riboflavin, also known as vitamin B2, to induce crosslinks in the collagen matrix of the corneal stroma. This approach depends on the photosensitizer effect of riboflavin, which, when activated by UVA light with wavelengths of 365 or 370 nm, produces reactive oxygen species that lead to crosslinks and change in the chemical properties of the corneal stroma. CXL has also emerged as a new line of management for corneal infections through improved collagen resistance and bactericidal effect due to intercalation between DNA and RNA causing nucleic acid damage when exposed to UV that eventually leads to rapid elimination of the infecting microorganism and reducing inflammation [3–5]. Experimental evidence suggests that riboflavin and UVA photochemical cross-linking increases the corneas' resistance to collagenase, trypsin, and pepsin and interferes with the toxic enzymatic digestion, which causes the corneal melting by proteases and prevents further thinning of the cornea [6, 7].

In the last decade, the evolving antimicrobial treatment resistance necessitate a second line or adjuvant treatment option for corneal infection. Meanwhile, more clinical trials have evaluated the role of CXL as an adjuvant therapy to conventional antimicrobial treatment. Only some studies have implemented the role of CXL monotherapy treatment without the confluent effect of conventional antimicrobial treatment. However, those clinical trials provided promising results in bacterial and fungal corneal infections. In particular, studies using CXL to treat *Fusarium* and *pseudomonas* keratitis revealed partial response to CXL [8–10].

To date, most studies that evaluated CXL in corneal infections have combined CXL with antimicrobial therapy; however, CXL as a monotherapy is still underestimated. This study aims to assess the efficacy of CXL as a monotherapy for managing infectious keratitis with similar severity in an experimental animal model caused by *Fusarium solani* and *Pseudomonas aeruginosa*.

## Methodology

This is a prospective comparative experimental study in rabbits, conducted in the experimental animal facility of the Research Institute of Ophthalmology, Giza, Egypt, from Aug 2021 to April 2022. All rabbits were middle-aged, white New Zealand, weighing approximately 1.400 to 2.250 kg. The Memorial institute of ophthalmic research Ethics Committee approved the experiment. All procedures followed the Institutional Guidelines and the Statement for the Use of Animals in Ophthalmology and Vision Research and observed the essential Animal Research: Reporting of In Vivo Experiments (ARRIVE) guidelines for animal research. The experimental study was conducted on 48 eyes of rabbits with the clear cornea. They were divided into three groups (A, B, and C), with sixteen eyes in each group; the sample size was decided based on the outcome measures. Healthy litter cage animals were included and provided with food, water, and veterinary supervision. Randomization and blinding were initially applied to minimize the confounder bias.

Two organisms were employed to cause infection. Group A served as a control group with eight rabbits' corneas injected with *Fusarium solani* (A_1_) and the other eight rabbit's corneas injected with *Pseudomonas aeruginosa* (A_2_). Group B (*n* = 16) corneas were injected with *Fusarium solani*. Group C (*n *= 16) corneas were injected with *Pseudomonas aeruginosa*.

1. Preparation of inoculum of *Pseudomonas aeruginosa*: The organism was subcultured on nutrient agar and incubated at 37C for 24–48 h. The inoculum was prepared by suspending 3–5 colonies in 5 ml sterile saline. The suspension was mixed for 5 s, and the optical density was read using a spectrophotometer. The suspension was adjusted using saline to an absorbance of 0.08–0.1 at 625 nm to obtain a bacterial stock suspension of approximately 1–2 × 10^8^ CFU/ml (McFarland standard). The suspension was diluted by transferring 0.2 ml of the stock suspension into 19.8 ml of sterile saline. This resulted in a suspension of approximately 1 × 10^6^ CFU/ml. Rabbits were injected with 0.1 ml of the adjusted inoculum suspension.
2. Preparation of inoculum of *Fusarium solani*: The inoculum suspension of *Fusarium solani* was prepared from a fresh mature (3–5 days old) culture incubated at 30^0^C on plain Sabouraud’s dextrose agar medium (SDA). The fungal colonies were covered with 1 ml of sterile distilled water, and the suspensions were made by gently suspending 3–5 colonies in 5 ml of sterile saline. The suspension was vortexed for 10 s, and the optical density was read using a spectrophotometer. The turbidity of the suspension was adjusted using saline to an absorbance of 530 nm to obtain a stock suspension of approximately 1.0 × 10^6^ CFU/ml. Rabbits were injected with 0.1 ml of the adjusted inoculum suspension.

During injection, all animals were anesthetized with intramuscular ketamine at 30 mg/kg and subcutaneous xylazine hydrochloride (Xyla-Ject, Adwia Pharmaceuticals Co, Cairo, Egypt) at a dose of 20 mg/mL. The dose was increased whenever needed. The respiratory rate and heart rate were monitored. Then topical anaesthesia using 0.4% benoxinate hydrochloride was administered. The cornea of one eye of each animal was inoculated with the microorganism.

**Fig. 1:**
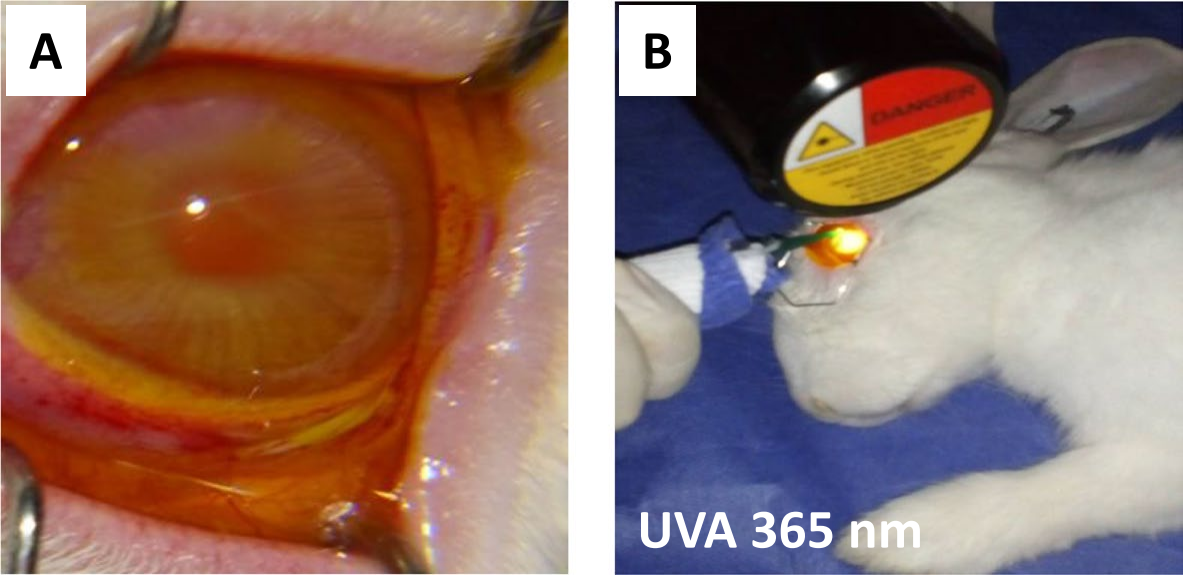
Collagen cross-linking method used in the study. **A**. Riboflavin photosensitizer solution was dropped onto the rabbit cornea every 5 min for 30 min before the irradiation. **B**. The cornea was irradiated with UVA-light (365 nm), intending an irradiance of 3.0 mW/cm^2^ for 15 min at a distance of 45 mm above the cornea

All animals in Group B and C received CXL treatment one week after inoculation of the organisms and after corneal abscess formation was confirmed. At the same time, animals in control group A were left untreated. Collagen cross-linking was done using OPTO XLINK corneal CXL device (Opto, São Carlos, Brazil). Using a standard aseptic technique, a scalpel blade completely scraped off the corneal epithelium around the abscess. Isotonic riboflavin photosensitizer solution (isotonic) containing 0.1% riboflavin-5-phosphate in dextran 2o% was dropped onto the cornea every 5 min for 30 min before the irradiation. During irradiation, as shown in (Fig. 1A), topical instillation of riboflavin was continued onto the eye every 3 min for another 15 continuous minutes of irradiation. The cornea was irradiated with UVA-light (365 nm), intending an irradiance of 3.0 mW/cm2 for 15 min at 45 mm above the cornea, as shown in (Fig. 1B). After irradiation, the eye was rinsed with a sterile saline solution.

Corneal scraping was done after CXL in the 1st, 2nd, 3rd, and 4th week to detect the organism's viability. Once a topical anaesthetic (lidocaine hydrochloride, 4%) had been applied, the corneal ulcer's leading edge and base were scraped with a Kimura spatula that had been sterilized in a flame. Two corneal scrapings were done as described in the mycotic ulcer treatment trial [11]. Materials obtained by the first and second scrapings were spot inoculated into the nutrient broth and incubated for 24 -48 h at 37 C. After incubation, 1 ml of bacterial broth was inoculated on nutrient agar; on the other hand, 1 ml of the fungal broth was inoculated on Sabouraud agar (SAB). The inoculum was spread on the plates uniformly using burned sterilized glass rod spreaders. The bacterial plates were incubated at 37c while the SDA media was incubated at 30 °C. And observed daily, colonies were counted as soon as possible after growth became visible. Colonies on the plates were usually counted by dividing the plates into equal sectors (1/2 up to 1/4). After counting one sector, the count was multiplied by the total number of sectors to estimate the whole plate CFU count. A quantitative analysis of microorganisms was performed. Treatment groups were compared according to the clinical and microbiological analysis score. Data management and statistical analysis were done. Numerical data were summarized as median, mean, and range.

Rabbits were mercifully sacrificed at the end of the 4th week, and the eyes were enucleated. Each animal’s cornea was removed by the same surgeon 1 mm close to the limbus, rinsed in standard saline solution, then fixed in phosphate-buffered 10% formaldehyde in labeled glass containers and sent for histopathological evaluation. As previously described, paraffin sections with 5 μm thickness were prepared and stained with Harris hematoxylin and eosin [12]. Collagen fibres were prepared and stained by Mallory’s trichome stain as described [13]. Corneal cellular infiltration has been elaborated by detecting Anti-CD20 (Rabbit Polyclonal Antibody, Thermo Scientific, Applied Biosystems, Invitrogen) with DAB (3,3′-Diaminobenzidine) chromogen counterstain for Immunohistochemistry technique as previously reported [14].

### Statistical analysis

Data were statistically described in frequencies (number of cases) and percentages. Comparison between different organisms was made using Chi-square (χ2) test. The exact test was used when the expected frequency was less than 5. For comparing between stages, the McNemar test was used. Two-way analysis of variance (ANOVA) was performed with subsequent Bonferroni tests. Two-sided p-values less than 0.05 was considered statistically significant. All statistical calculations were done using the computer program IBM SPSS (Statistical Package for the Social Science; IBM Corp, Armonk, NY, USA) release 22 for Microsoft Windows.

## Results

**Fig. 2:**
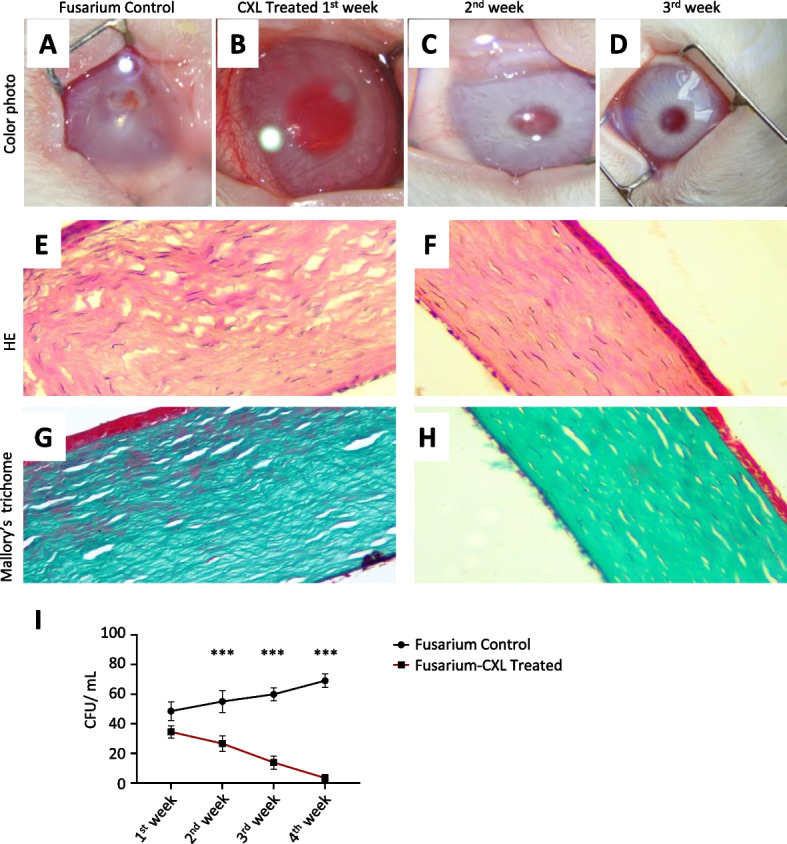
CXL as a monotherapy in the management of corneal infection caused by *Fusarium solani.* **A**, **B**, **C**, **D** Colour photographs of representative rabbit's corneas after *Fusarium* infection; image obtained in the untreated control group A1 at 1st week and for CXL-treated group B at 1st, 2nd 3rd week follow up. **E**, **F**, **G**, **H** Histopathological section of cornea-scleral rim stained by hematoxylin/eosin and Mallory’s trichome (200 × mag) shows separated, disrupted collagenous lamellae with a waving appearance and cellular infiltration in group A1 Fusarium control [left panel: **E**, **G**] while the well-formed corneal structure and complete healing with intact epithelium and substantia propria that forms the main bulk of the cornea in CXL treated group [right panel: **F**, **H**]. **I** Time course CFU/ml of samples from untreated controls (black line) and CXL treated (red line). Data are presented as mean and Std. Asterisks represent significant differences [**P ≤ 0.01, two-way analysis of variance (ANOVA) with subsequent Bonferroni test]

In *Fusarium solani* group, By the 3rd week of infection with the same *Fusarium* organism under similar conditions, the Control untreated cornea Group A1 shows severe corneal destruction with extensive inflammation (Fig. 2A). While CXL-treated group B complete healing, no active ulceration nor corneal infiltrates with normal corneal reflex maintains normal corneal examination (Fig. 2 B, C, D). The histopathological section done after 4th week of infection on the cornea-scleral rim shows separated, disrupted collagenous lamellae with a waving appearance and cellular infiltration in group A1 *Fusarium* control (Fig. 2E, G), while the well-formed corneal structure and complete healing with intact epithelium and substantia propria that forms the main bulk of the cornea in CXL treated group (Fig. 2 F, H).

A statistically significant reduction in the number of colony-forming units (CFU) in the *Fusarium* group following CXL is shown in (Fig. 2I). No growth existed in any samples at the end of the 4th week. There was a statistically significant difference in the number of CFU between group B and the control group (*p* < 0.001) (Table 1, Fig. 2I).

**Table 1:** Colony forming units count of *Fusarium solani* in the untreated control group A_1_ and CXL-treated group B

	*Fusarium* Control (*n* = 8)		*Fusarium* Treated (*n* = 16)				
	Mean	St.Dev	Mean	St.Dev	t ratio	df	*P* value
1st week	48.57	6.27	34.50	4.11	5.212	13	0.001
2nd week	55.00	7.48	26.60	5.32	7.096	9	< 0.001
3rd week	59.86	4.38	13.83	4.36	18.95	11	< 0.001
4th week	69.00	4.65	3.40	2.30	28.81	10	< 0.001

**Fig. 3:**
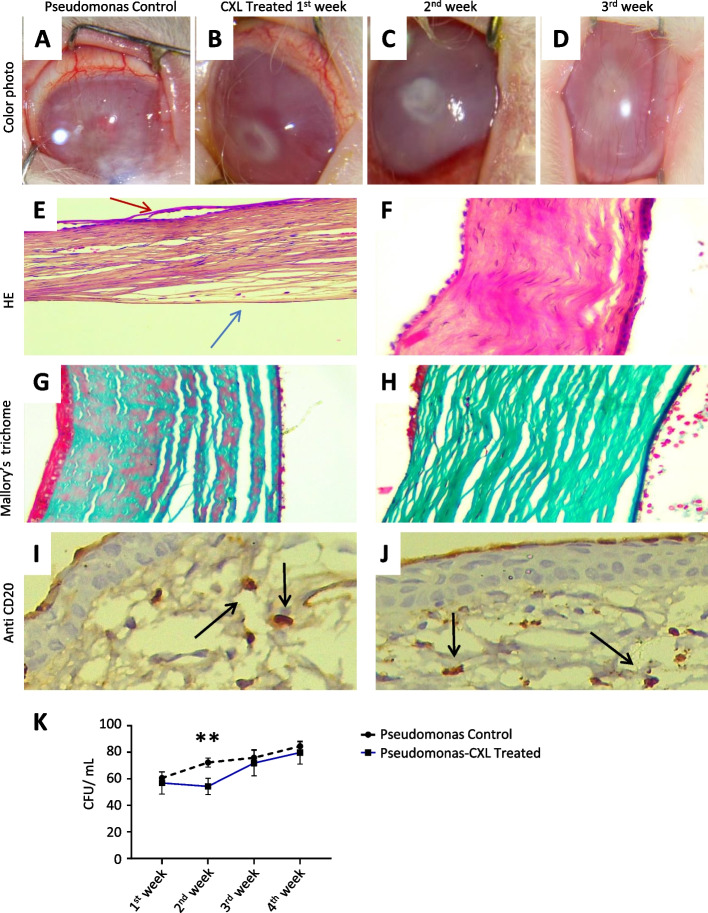
CXL as a monotherapy in the management of corneal infection caused by *pseudomonas aeruginosa.* **A**, **B**, **C**, **D** Colour photographs of representative rabbit's corneas after *pseudomonas* infection; image obtained in the untreated control group A1 at 1st week and for CXL-treated group B at 1st, 2nd 3rd week follow up. **E**, **F**, **G**, **H** Histopathological section of cornea-scleral rim stained by hematoxylin/eosin and Mallory’s trichome (200 × mag) shows corneal stromal thinning, defects, detachment of epithelium (red arrow), Descemet’s membrane separation (Blue arrow), and widely separated thin collagenous lamellae [left panel: **E**, **G**]. While there is less corneal thinning In group C, there is a loss of part of the epithelium (corneal ulcer), irregular Bowman's layer, and stromal infiltrates [right panel: **F**, **H**]. **I**, **J** immunohistochemistry section showing immunopositive staining with anti CD20 positive cells for B cell infiltrates (brown staining, black arrow); [**I**] for control, [**J**] for CXL-treated group C; DAB chromogen used as a counter stain. **K** Time course CFU/ml of samples from untreated controls (black line) and CXL treated (red line). Data are presented as mean and Std. Asterisks represent significant differences [***P ≤ 0.001, two-way analysis of variance (ANOVA) with subsequent Bonferroni test]

In the *Pseudomonas* group, At the 1st week of infection with the same organism under similar conditions, there was aggressive corneal edema and ulceration with extensive inflammation (Fig. 3A). No improvement was detected in any of CXL-treated group C, nor healing over the four weeks follow-up period. Instead, there were active ulceration, and corneal infiltrates with impending corneal melting (Fig. 3 B, C, D). Histopathological section done after 4th week of infection on the cornea-scleral rim stained by hematoxylin/eosin and Mallory’s trichome shows corneal stromal thinning, defects, detachment of epithelium, Descemet’s membrane separation and widely separated thin collagenous lamellae (E, G). While there is less corneal thinning in CXL-treated group C, there is an active corneal ulcer, irregular Bowman's layer, and stromal infiltrates (Fig. 3 F, H).

To further investigate that CXL has no immune suppression effect in severe pseudomonas infection as a possible cause of rapid progression after an initial decline, we performed an immunohistochemistry analysis of pseudomonas-infected corneas in 4th week after infection. Both control and CXL-treated corneas had similarly subepithelial CD20 + B cell infiltrates (Fig. 3 I, J).

There was a statistically significant reduction in the CFU at the end of the first week after CXL. However, there was regrowth in all samples afterward. All 16 samples showed countable growth during the subsequent follow-ups. There was no difference between the number of CFU in the untreated control group A2 and CXL-Treated Group C (*p* = 0.17) (Table 2, Fig. 3 K).

**Table 2:** Colony forming units count of *Pseudomonas aeruginosa* in the untreated control group A_2_ and CXL-treated group C

	*Pseudomonas* Control (*n* = 8)		*Pseudomonas* Treated (*n* = 16)				
	Mean	St.Dev	Mean	St.Dev	t ratio	df	*P* value
1st week	60.63	4.44	56.88	8.37	1.12	14.00	0.28
2nd week	72.25	3.45	54.25	6.09	7.27	13.00	< 0.01
3rd week	75.88	6.17	71.88	9.64	0.99	14.00	0.34
4th week	84.50	3.34	79.75	8.73	1.44	14.00	0.17

## Discussion

CXL has been regularly used in refractory and non-responding infectious keratitis as an adjuvant to antimicrobial therapy [15]. In this study, we presented the favourable results of CXL as a monotherapy as a first line in the treatment of *Fusarium solani.* We also investigated the efficiency of *Pseudomonas aeruginosa* keratitis models experimentally induced in rabbits’ eyes with equal severity with unfavourable results.

This study shows that the antimicrobial properties of riboflavin and UVA light (CXL) were effective as monotherapy in *Fusarium* keratitis. The antimicrobial effect started to appear clinically in the 3rd week following CXL, with no growth in 31.3% of samples and an absence of growth in all samples (100%) by the 4th week following CXL. Although some in vitro studies reported little value of CXL on Candida albicans and *Fusarium solani* [16], many other clinical studies reported that CXL significantly reduced fungal activity in *Fusarium* keratitis [8, 17]. We reason for the multiple mechanisms of action of CXL in viable corneas, which substantially differ from the in vitro environment. Moreover, Özdemir et al. concluded that the association of CXL with topical medical treatment was influential in cases of fusarium, candida, and Aspergillus-infected keratitis, especially in the early course of the disease [18, 19].

The value of CXL is further highlighted by the fact that more than 70 species of fungal genera cause fungal keratitis [20]. Subsequently, the response to medical treatment might differ. Notably, a large prospective clinical trial done in India to evaluate CXL in fungal keratitis revealed no additional benefits for adjuvant CXL in managing moderate fungal ulcers. Conversely, it may decrease visual acuity and worsen the prognosis [21]. This can be explained by the fact that the subjects of this study were all from India, and infection was attributed to agricultural exposure and not contact lens wear like those of developed countries.

Although the in vitro studies strongly suggested that UV, when combined with riboflavin, can eradicate Staphylococcus aureus, methicillin-resistant S aureus (MRSA), and *Pseudomonas aeruginosa* in agar plates [22], this cannot be fully proved in the setting of clinical trials. It can be explained that in vitro, there is no enzymatic digestion of the stroma as an additional factor to corneal destruction [23].

This study found that CXL had some deloading effect on *pseudomonas* in the first week following treatment. This notion has been evidenced by other clinical trials done by Prajna et al., in which CXL failed to eradicate bacterial keratitis. Still, it reduces culture positivity and decreases the complication progression, particularly if applied to the earlier course of the disease [23]. To exclude that CXL had immune suppression impact in severe pseudomonas infection, which may explain rapid progression after an initial decline. We found that both control and CXL-treated corneas had similar subepithelial CD20 + B cell infiltrates. The present study also concluded that CXL, as a monotherapy, might not be effective in such cases but helps the deloading of the organisms (pseudomonas) without eradication. We also noted that corneal thinning with probability for corneal melting or need for urgent keratoplasty is lesser in CXL-treated corneas, which can be explained by the suppression of proteolytic enzymes that are released from both organism and leukocytes [24, 25]. Likewise, combining medical treatment with CXL in these cases is still recommended.

One of the limitations of the current study is that the study was done on the same pathogens under similar conditions that did not correlate with the wide variety of infective species and the effect of the environment. We did not compare the impact of antimicrobial alone to monotherapy CXL in predicting the final outcome, so we recommend a large prospective comparative clinal trial to evaluate CXL as a mono or adjuvant therapy for infective corneal ulcer.

## Conclusion

Collagen cross-linking is promising monotherapy and alternative treatment in managing infective keratitis caused by *Fusarium solani* but is less effective in *Pseudomonas aeruginosa* as monotherapy. Evidence in a large clinical trial is still recommended for other pathogens and for evaluating CXL as a mono or adjuvant therapy for infective corneal ulcers.

## Data Availability

The data supporting the findings of this study are available within the article and for further details on requests from the corresponding author.

## Abbreviations

ARRIVE: Animal Research: Reporting of In Vivo Experiments
CXL: Collagen Cross Linking
CFU: Colony Forming Units
RCULA: Responsible Care and Use of Laboratory Animals
UVL: Ultraviolet Light
